# Supplementary material for: Introduction of Reversible Urethane Bonds Based on Vanillyl Alcohol for Efficient Self-Healing of Polyurethane Elastomers
Source: Molecules. 2019 Jun 12;24(12):2201. doi: 10.3390/molecules24122201 (PMC6637387; doi:10.3390/molecules24122201)
Supplement: Supplementary file 1 [file molecules-24-02201-s001.pdf]

Supporting Information

Introduction of reversible urethane bond based on vanillyl alcohol for efficient self-healing of polyurethane

Dae-Woo Lee †, Han-Na Kim †, and Dai-Soo Lee *

Division of Semiconductor and Chemical Engineering, Chonbuk National University, Baekjedaero 567, Jeonju 54896, Republic of Korea; dwlee2310@hanmail.net (D.W.L.); hnk07@hanmail.net (H.N.K.)
† Contributed equally to this work
* Correspondence: daisoolee@jbnu.ac.kr; Tel.: +82-63-270-2310

Table S1. Thermal properties of synthesized PUs.

Sample code	Temperature (℃)			
	5% T_d *[1]	10% T_d *[1]	T_{gs} *[2]	T_{flow} *[2]
**Control PU**	**311**	**331**	**-29**	**148**
**VA10**	**303**	**317**	**-31**	**133**
**VA20**	**301**	**316**	**-25**	**129**
**VA30**	**300**	**315**	**-17**	**129**
**VA40**	**298**	**315**	**-9**	**125**
**VA40-5**	**297**	**313**	**-14**	**131**
**VA40-10**	**295**	**313**	**-20**	**127**

*[1] T_d was measured by TGA

*[2] T_{gs} and T_{flow} were measured by DMA

Table S2. Relaxation time and activation energy of synthesized PUs.

Sample code	Relaxation time (s)					Activation energy (kJ/mol)
	120 ℃	130 ℃	140 ℃	145 ℃	150 ℃	
Control PU	-	-	539	241	98	247.6
VA10	-	220	71	-	33	134.6
VA20	-	315	102	-	38	149.9
VA30	-	380	120	-	45	151.2
VA40	-	614	162	-	55	171.1
VA40-5	408	147	65	-	-	124
VA40-10	294	114	59	-	-	108.5

Table S3. Tensile strength and healing efficiency of synthesized PUs at 140 °C for 30 min.

Sample code	Tensile strength (MPa)		Healing efficiency (%)
	Before healing	After healing	
Control PU	25.5	17.7	69.3
VA10	29.5	17.8	60.5
VA20	32.8	16.6	50.7
VA30	35.7	15.3	42.9
VA40	37.2	12.1	32.4
VA40-5	18.4	16.3	88.4
VA40-10	14.9	14.4	96.5

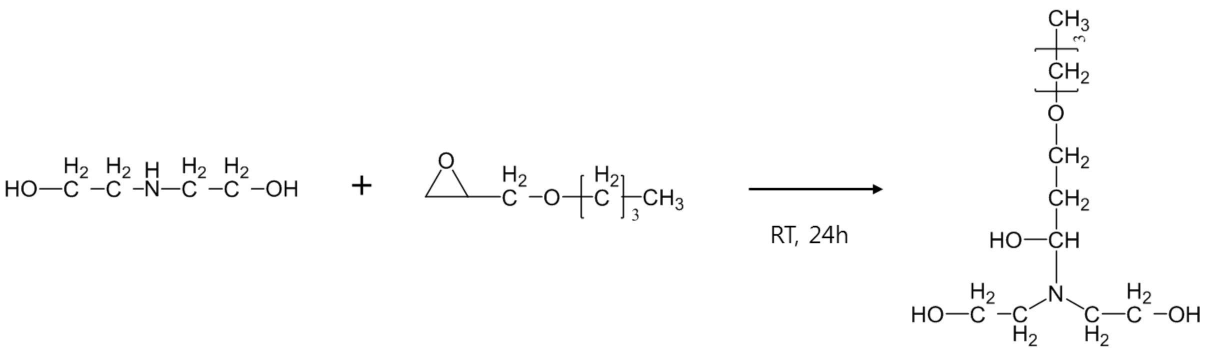

Figure S1. Synthesis of m-CE.

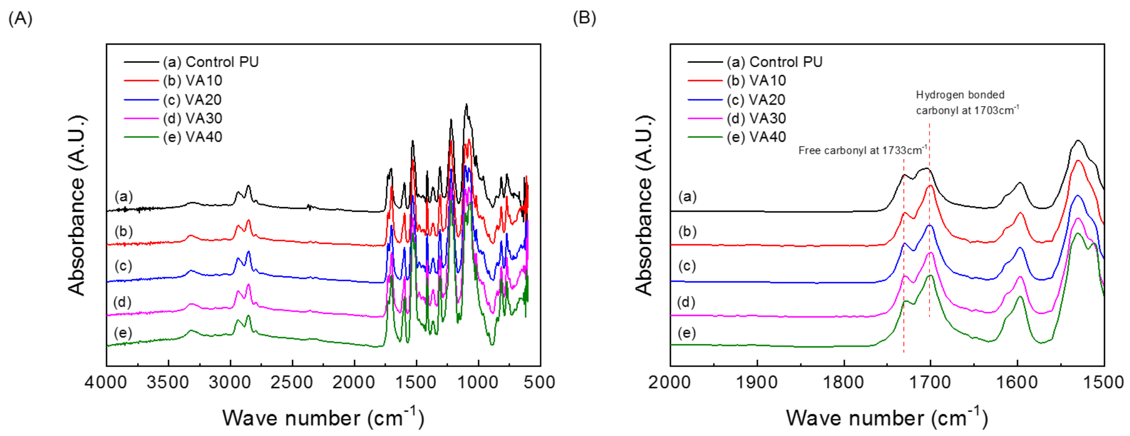

Figure S2. FT-IR spectra of VA-based PUs.

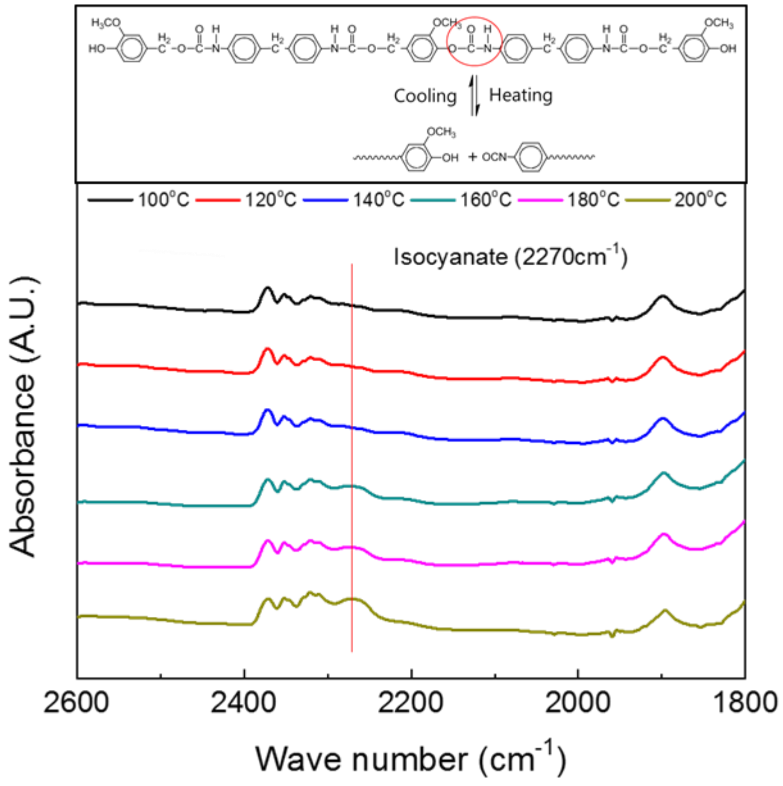

Figure S3. FT-IR spectra of model structure for reversible urethane bond at elevated temperature.

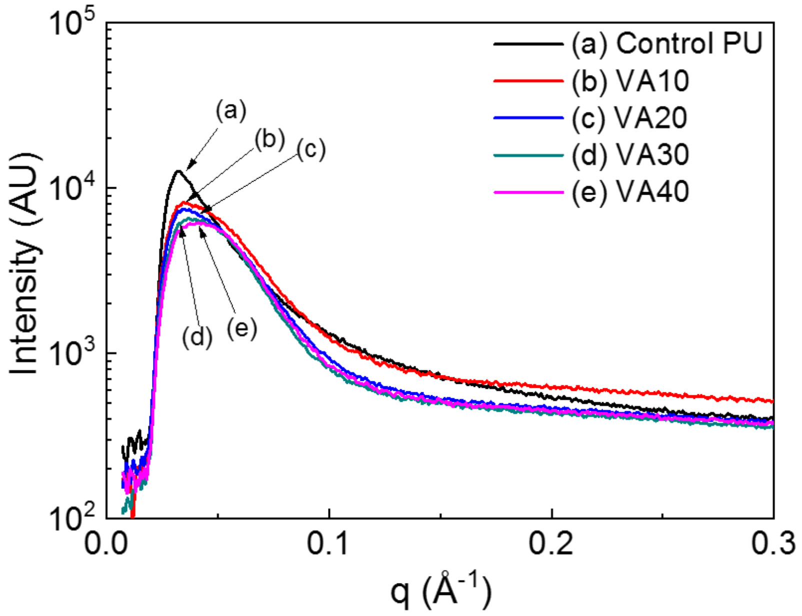

Figure S4. SAXS profile of VA-based PUs.

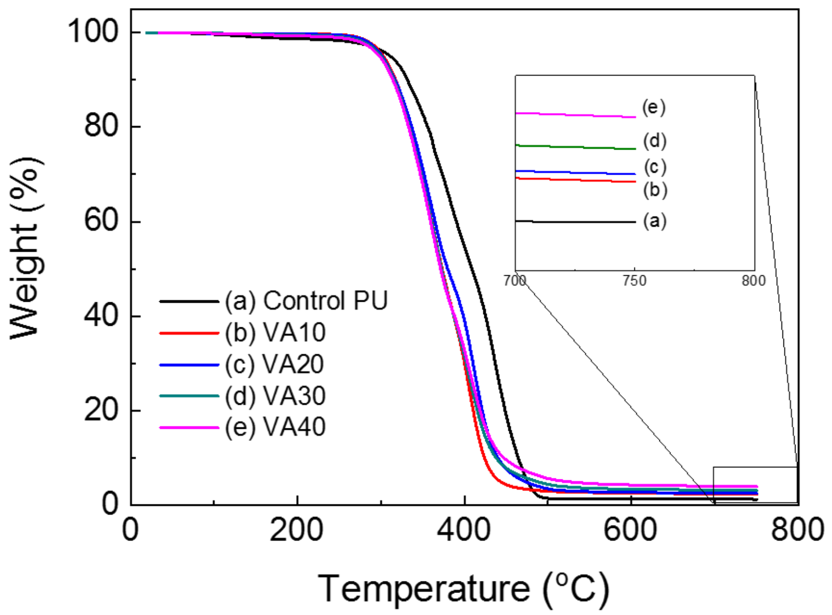

Figure S5. TGA thermograms of VA-based PUs.

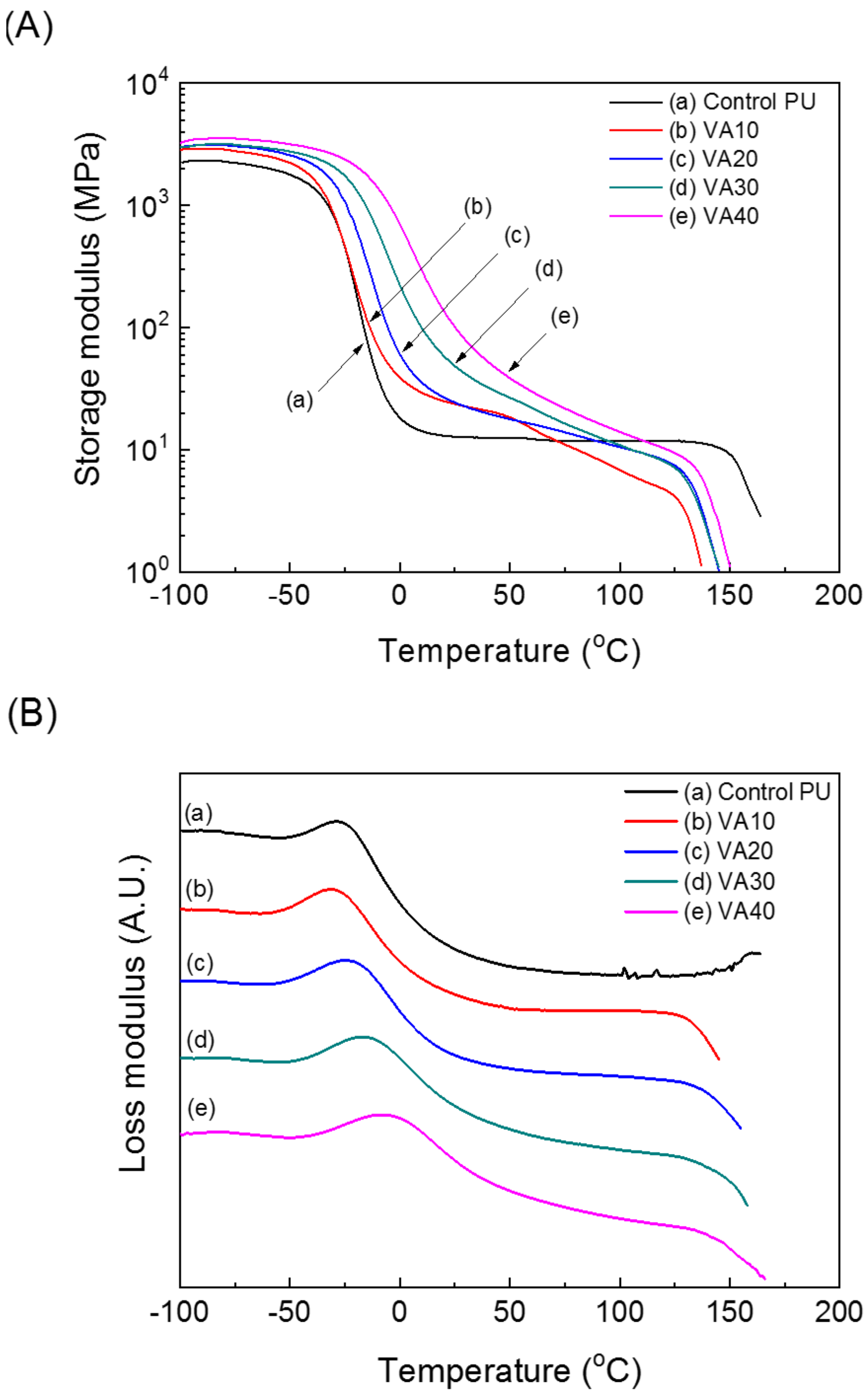

Figure S6. DMA thermograms of VA-based PUs.

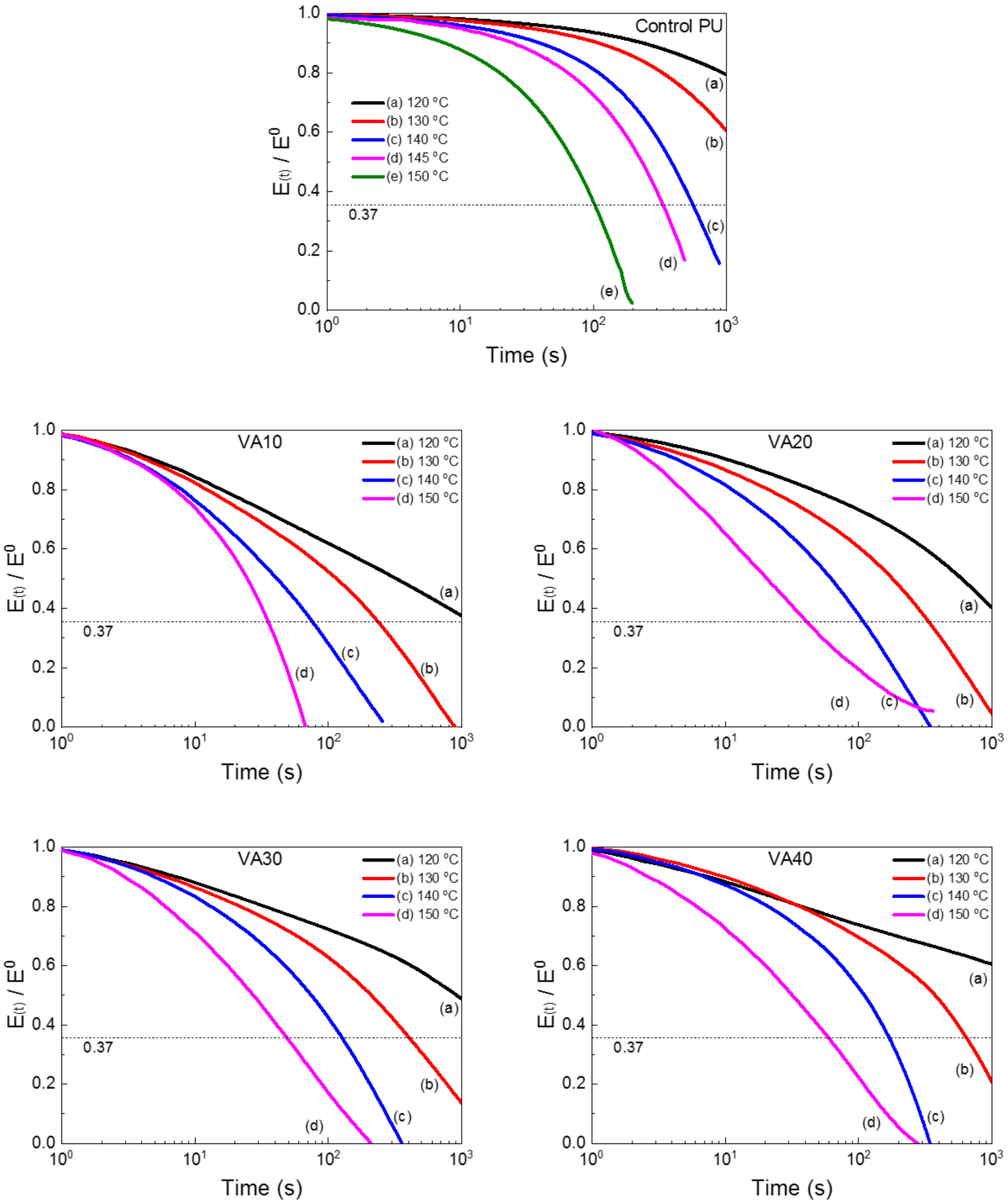

Figure S7. Stress-relaxation curves of the VA-based PUs at various temperatures.

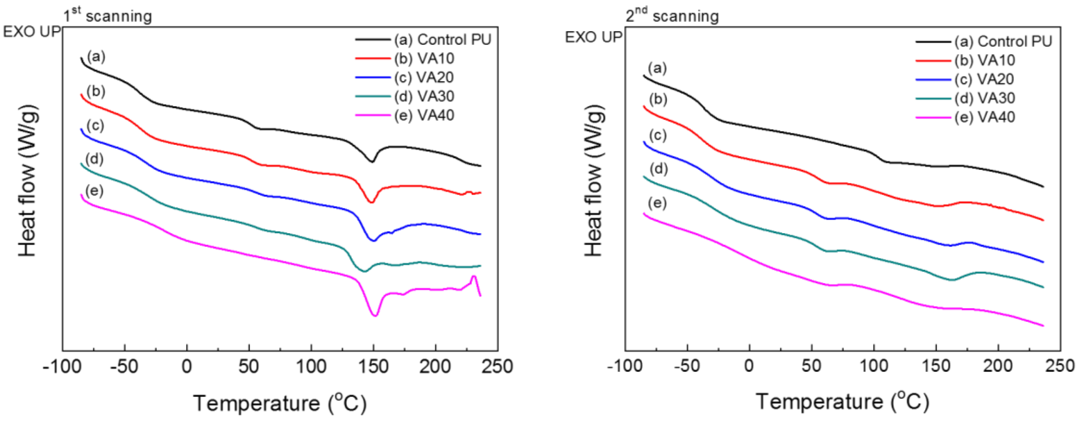

Figure S8. DSC thermograms of VA-based PUs.

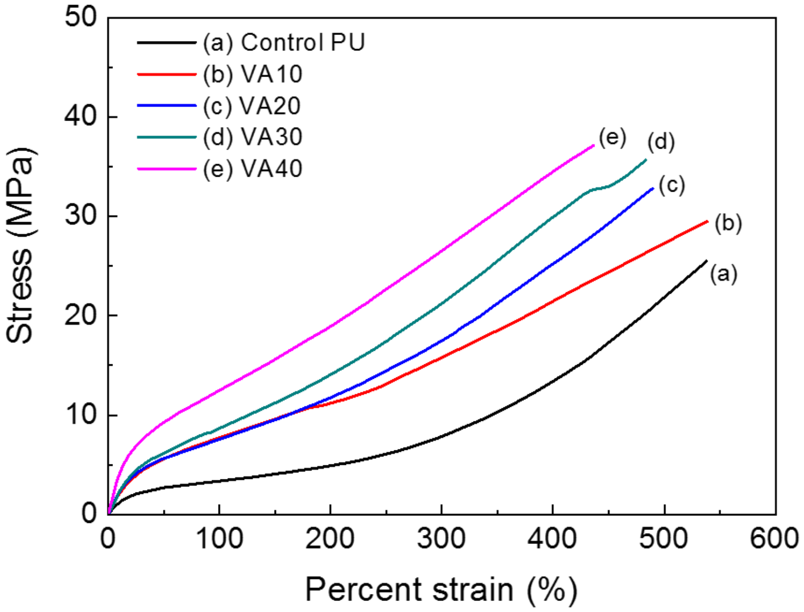

Figure S9. Stress–strain curves of VA-based PUs.

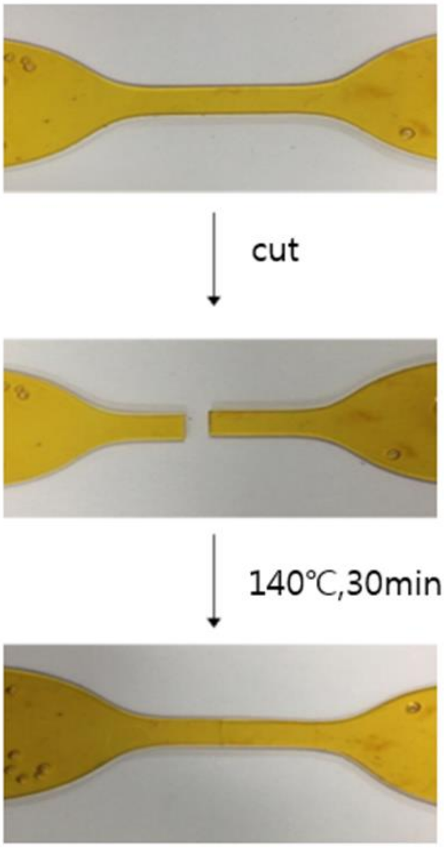

Figure S10. Images of self-healing test specimens for VA40-10.

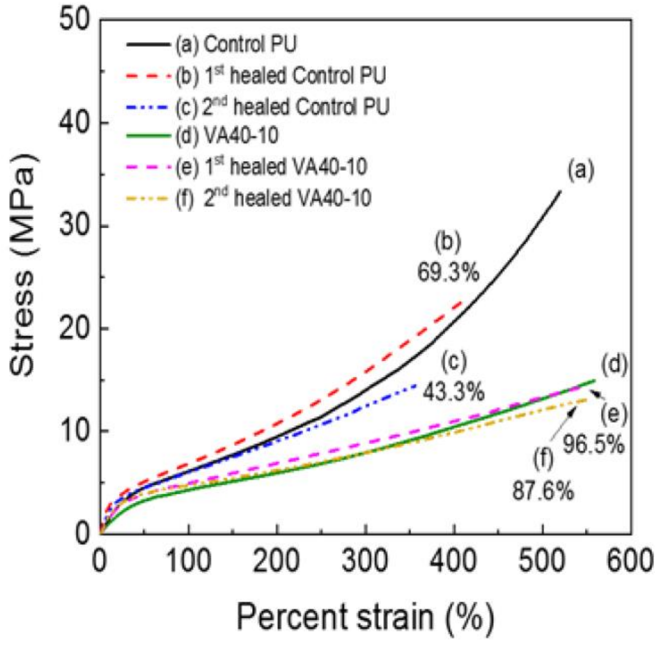

Figure S11. Tensile properties of PUs after repeated healing at 140 ℃.

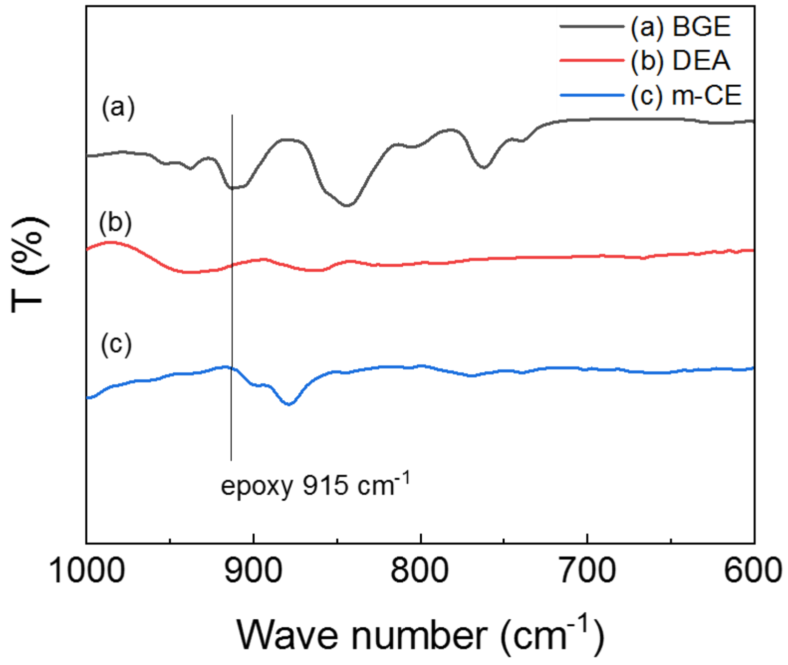

Figure S12. FT-IR spectra of DEA, BGE and m-CE.

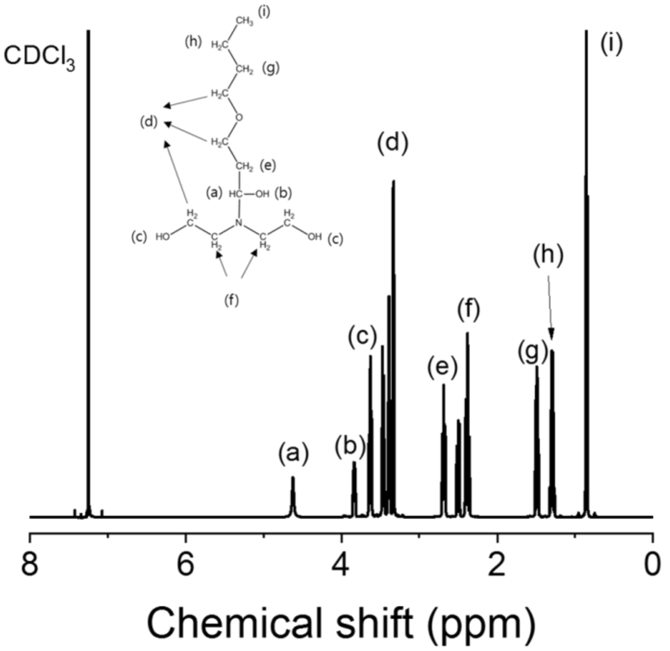

Figure S13. NMR spectrum of m-CE.
